# A Comparative Study of the Improved Negative-Pressure Drainage Tube and the Penrose Silicone Drainage Tube: Which Is More Beneficial for Wounds from Deep Infections Healing? A Rabbit Model Study

**DOI:** 10.1007/s43465-025-01601-4

**Published:** 2025-10-27

**Authors:** Jingwen Jia, Ziyan Wei, Yanan Chen, Shuwei Chen, Yingping Ma, Xuewen Kang

**Affiliations:** 1 Department of Orthopaedics, Lanzhou University Second Hospital, No. 82 Cuiyingmen, Chengguan District, Lanzhou, 730000 Gansu People’s Republic of China; 2 Gansu Provincial Hospital, Lanzhou, 730000 Gansu People’s Republic of China

**Keywords:** Model of infection, Negative-pressure wound therapy, Wound drainage

## Abstract

**Background:**

Postoperative orthopedic wounds frequently lead to deep infections, and conventional drainage tubes exhibit inadequate drainage capacity. This study aimed to assess the efficacy of an innovative and improved negative-pressure wound irrigation drainage tube in enhancing infection control and facilitating wound healing.

**Methods:**

Forty New Zealand rabbits were randomly assigned to five groups: A (control), B (abscess model), C (Penrose drain), D (improved negative-pressure wound irrigation drainage tube with drainage), and E (improved negative-pressure wound irrigation drainage tube with drainage and irrigation). A deep wound infection model was established, and its success was assessed using Gram staining and mass spectrometry. Daily weight, temperature, and drainage volume were recorded for each rabbit group. In vitro cellular experiments were performed to assess the biocompatibility of the materials. Histological examinations were conducted on postoperative day 14 to evaluate wound healing.

**Results:**

Bacterial culture demonstrated the consistent presence of primary pathogenic bacteria in all groups, confirming the successful establishment of the model. Following surgery, all rabbit groups showed a consistent increase in body weight with no significant variation (*P* > 0.05). On day 14, the average skin temperature of Group E was significantly lower than that of the other groups (*P* < 0.001). The average total drainage volumes on day 14 were 9.03 ± 0.60 ml for Group C and 10.49 ± 0.99 ml for Group D, indicating significantly higher drainage in Group D than in Group C (*P* < 0.05). In vitro experiments demonstrated that the product had no adverse effect on the viability and proliferation of fibroblast cells. Histological analyses indicated that muscle tissue structure was normal in Group A, exhibited cell loss with inflammatory cell infiltration in Group B, displayed slight abnormalities with mild fibrosis in Group C, showed mild irregularities with a relatively organized arrangement of muscle cells in Group D, and remained essentially normal in Group E.

**Conclusion:**

The improved negative-pressure wound irrigation drainage tube demonstrated distinct advantages over the conventional Penrose silicone drain in controlling inflammation, optimizing wound treatment, and promoting wound healing in deep wound infections.

## Introduction

Deep wound infection is a prevalent and severe postoperative complication that, if untreated, can exacerbate a patient’s condition and result in a poor prognosis. Previous studies suggest that postoperative infections in orthopedic surgeries contribute to approximately 40% of hospital-acquired infections, primarily because of the frequent use of implants in orthopedic procedures, which significantly elevates the infection risk [1, 2]. Orthopedic patients are often older and require prolonged surgeries, and extended postoperative immobilization. Consequently, the prevention of deep wound infections has emerged as a complex clinical challenge and a prominent global research focus [3, 4]. Currently, these infections are managed using debridement, antibiotics, and various treatment modalities. However, these interventions are plagued by issues such as inadequate debridement and the emergence of antibiotic resistance, leading to suboptimal clinical outcomes [5–7]. The pathogenesis of infections often involves fluid accumulation. Ensuring complete drainage of the fluid, along with its electrolytes and proteins, can stabilize the osmotic pressure and swelling gradient at the wound site [8]. Consequently, postoperative irrigation drainage has become a widely adopted novel debridement approach in the clinical setting.

Vacuum-assisted closure (VAC), also known as negative-pressure wound therapy (NPWT), was initially introduced by Professor Fleischmann in Germany in the 1990s [9]. This approach involves applying negative pressure to wounds to efficiently extract pus or secretions, promote the growth of granulation tissue, and expedite wound healing [10]. Widely adopted in managing chronic, subacute, and acute wounds, including diabetic ulcers, organ/cavity fistulas, and orthopedic wounds, NPWT has consistently demonstrated positive therapeutic outcomes [11, 12]. Studies indicate that NPWT significantly reduces the bacterial load, prevents infection-induced tissue necrosis, and stimulates early granulation tissue formation compared to traditional gauze dressings [13]. In the current clinical practice, NPWT commonly uses silicone tube drainage. Nonetheless, standard negative-pressure drainage tubes, typically single-lumen and soft in texture, present challenges in deep wound insertion owing to their susceptibility to soft tissue blockages, causing incomplete drainage, potentially elevating postoperative hematoma risk, and impeding wound healing [14].

To address these issues, we developed an improved negative-pressure wound irrigation drainage tube, which successfully obtained a national invention patent (Patent No. ZL201510429543.X). Compared with traditional drainage tubes, the improved negative-pressure wound irrigation drainage tube offers the following advantages: (1) it is made of silicone with excellent biocompatibility, minimizing rejection; (2) multifunctional, reducing the number of drainage tubes for easier placement and management; and (3) features a specially designed square support plum blossom-shaped tube opening, ensuring smooth drainage and reduced risk of blockage. To verify the precise clinical advantages and application value, we compared its drainage effects with that of the traditional Penrose silicone drainage tube in a deep wound infection model in New Zealand rabbits. This study aimed to provide evidence supporting its future clinical use and offer a new and superior choice for NPWT.

## Methods

### Design and Production of the Improved Negative-Pressure Wound Irrigation Drainage Tube and Pressure Measurement of Negative-Pressure Suction Balls

#### Design Details of the Improved Negative-Pressure Wound Irrigation Drainage Tube

The enhanced negative-pressure wound irrigation drainage tube was designed with a multilumen structure, combining the features and functions of various existing tubes into one unit, minimizing the need for multiple drainage tubes for simplified placement and management. It consists of an internally inserted drainage tube and an externally connected tube as its primary components.

**Fig. 1:**
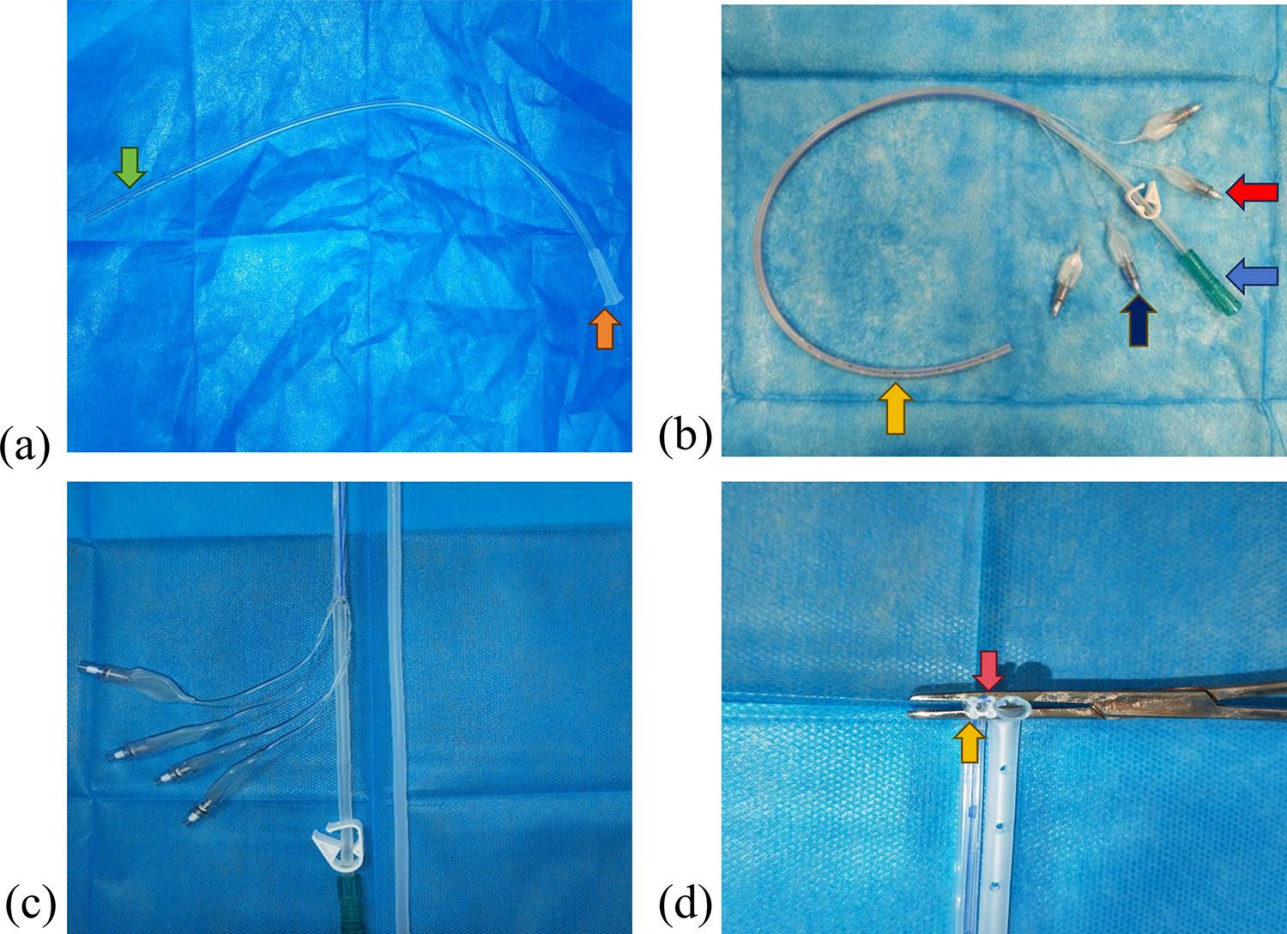
**a** Schematic diagram of the Penrose drainage tube, with the green arrow indicating the drainage hole and the orange arrow indicating the main drainage tube; **b** schematic diagram of the improved negative-pressure wound irrigation drainage tube, with the orange arrow indicating the drainage hole, blue arrow indicating the external portion of the main drainage tube, the red arrow indicating the external portion of the flushing tube, and the purple arrow indicating the external portion of the guidewire implantation tube; **c** comparison diagram of the two drainage tubes; **d** cross-sectional comparison diagram of the two drainage tubes, with the orange arrow indicating the internal portion of the improved negative-pressure wound irrigation drainage tube guidewire implantation tube, and the red arrow indicating the internal portion of the flushing tube

The internally inserted drainage tube is constructed as a cohesive unit comprising the main drainage tube, flushing tube, and guidewire implantation tube aligned longitudinally. Its cross-section resembles a four-petal plum blossom, with the main drainage tube situated at its center, while the flushing and guidewire implantation tubes are positioned around the perimeter of the main drainage tube. Numerous drainage apertures are situated within the concave groove of the external wall of the internally inserted drainage tube and linked to the main drainage tube to enhance drainage efficiency. The external connection tube included an independent external drainage tube, two external flushing tubes, and two external guidewire implantation tubes, each extending from the main drainage tube, flushing tube, and guidewire implantation tube at the outer end of the internally inserted drainage tube. The body of the external guidewire implantation tube can be fitted with an internal metallic wire to enhance the rigidity of the drainage tube and facilitate easier fixation (Fig. 1).

#### Production of the Product

This product has been entrusted to PureTech Technology Co., Ltd. for production.

#### The Measurement of Negative-Pressure Suction Balls

**Fig. 2:**
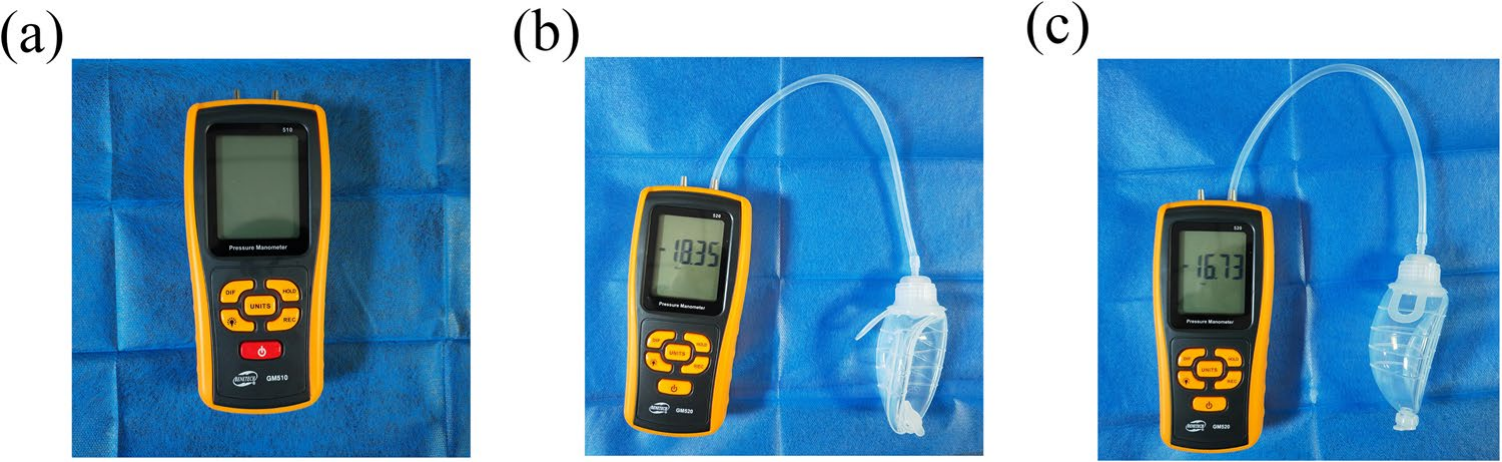
**a** Schematic diagram of the electronic manometer**. b**–**c** Connecting the negative-pressure suction ball to the electronic manometer to measure negative pressure (kpa)

To determine the pressure exerted by the negative-pressure suction ball under various deformations, we randomly selected three suction balls from an identical batch. The balls were attached to an electronic pressure gauge, and alterations in the negative pressure were recorded from full compression to 24 h (Fig. 2). Subsequently, a graphical representation of gradual pressure dynamics was generated.

### Experimental Animals and Grouping

Forty male New Zealand White rabbits aged 6 months and weighing 2.40–2.58 kg, were procured from the Lanzhou Veterinary Research Institute of the Chinese Academy of Agricultural Sciences (license number SCXK (Gan) 2020-0002), Lanzhou, China. These rabbits were accommodated in standard animal facilities, provided with ad libitum access to pellet feed, and housed at a room temperature of 19–22 °C with controlled lighting, regular disinfection, and proper ventilation. The rabbits were randomly allocated to five groups (A, B, C, D, and E), each comprising eight individuals. Group A served as the control without any specific intervention, Group B was the abscess model group subjected to abscess induction, Group C received a Penrose drainage tube for drainage after abscess induction, Group D underwent drainage using the modified negative-pressure wound irrigation drainage tube after abscess induction, and Group E underwent drainage and irrigation using the modified negative-pressure wound irrigation drainage tube with physiological saline after abscess induction. All animal procedures complied with the National Institutes of Health guidelines for the Care and Use of Laboratory Animals and were approved by the Animal Care and Use Committee of Lanzhou University Second Hospital (Approval No.: D2023-210) (Table 1).

**Table 1:** Experimental animal grouping and intervention measures

Number	Group name	Sample size	Intervention measures
A	Control group	8	No specific treatment
B	Abscess model group	8	Deep wound infection model
C	Penrose drainage tube group	8	Modeling of deep wound infection with Penrose drainage tube insertion for drainage
D	Improved negative-pressure wound irrigation drainage tube drainage-only group	8	Modeling of deep wound infection with insertion of the improved negative-pressure wound irrigation drainage tube for drainage
E	Improved negative-pressure wound irrigation drainage tube drainage and irrigation group	8	Modeling of deep wound infection with the insertion and irrigation drainage of the improved negative-pressure wound irrigation drainage tube

### Establishment of a Deep Wound Infection Model in New Zealand Rabbits and Insertion of the Drainage Device

**Fig. 3:**
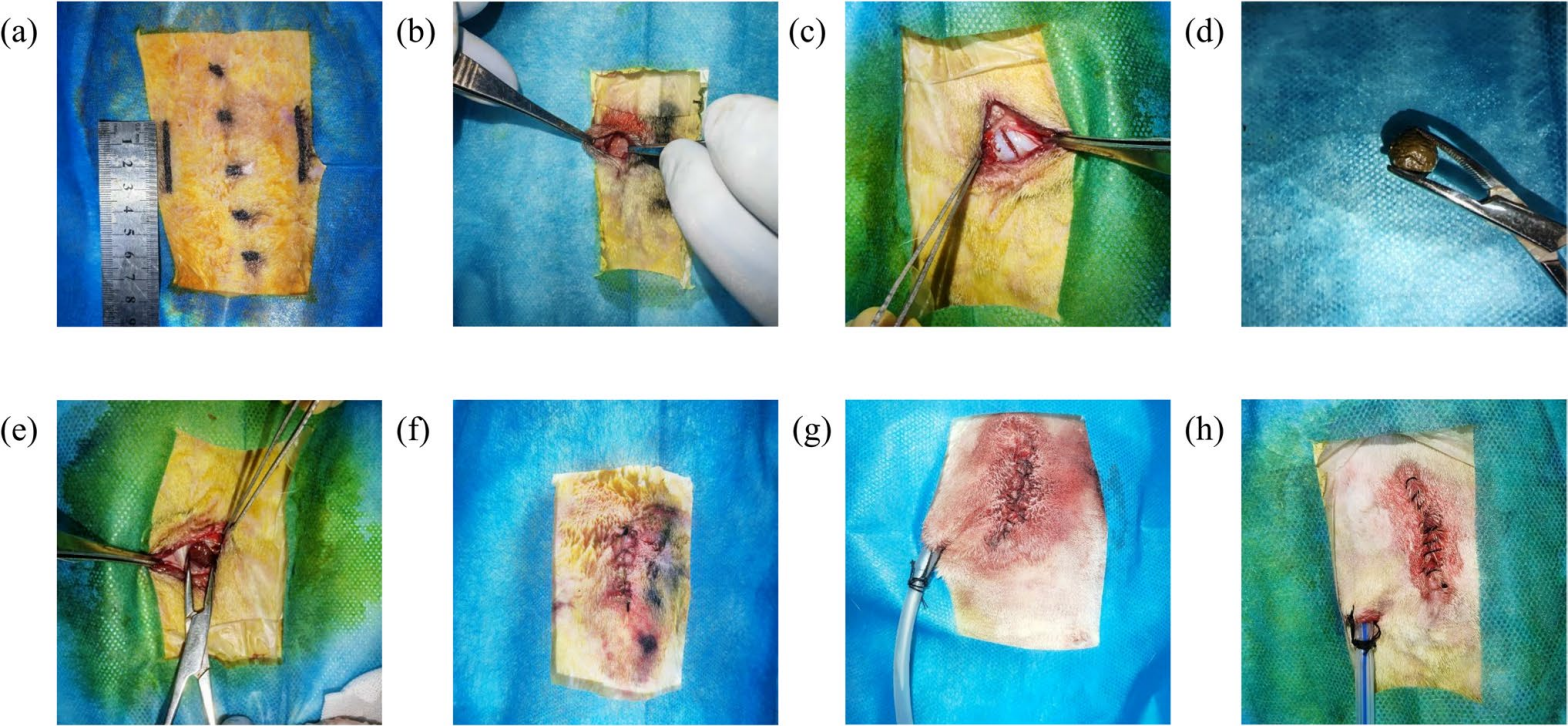
Procedure steps, **a** prepare skin disinfection to determine the wound location; **b** incise the skin; **c** cut the muscle; **d** diagram of rabbit feces; **e** embed the feces; **f** suture the wound; **g** insert a Penrose drainage tube; **h** insert a modified negative-pressure wound irrigation drainage tube

The rabbits in group A did not receive any special treatment. Group B rabbits were weighed and anesthetized via a marginal ear vein with a 3% pentobarbital sodium solution (30 mg/kg), positioned prone with limbs immobilized, and the surgical area on the back was prepared aseptically. Symmetrical 3 cm incisions were made 2.5 cm bilaterally from the rabbit’s spine. The skin was incised with a scalpel, the subcutaneous tissue was dissected bluntly, the muscles were incised, and two fresh fecal samples from New Zealand white rabbit were embedded, followed by wound closure. In Group C, a modeling procedure similar to that in Group B was conducted, and a Penrose drainage tube was inserted 2 cm deep into the wound from a 2 cm lateral incision beneath the parallel wound border on the rabbit’s back before wound closure. Groups D and E followed the same procedure, but with a modified negative-pressure wound irrigation drainage tube (patent number: ZL201510429543.X) inserted 2 cm deep into the wound from an incision made 2 cm laterally below the parallel wound border on the rabbit’s back before closure. Group E animals received 500 ml saline irrigation daily after abscess formation until wound healing. All the rabbits had postoperative wounds covered with gauze to prevent air leakage. Each drainage tube in Groups C, D, and E was connected to a negative-pressure suction ball, flattened, and maintained under negative pressure. The animals were monitored for normal breathing and heartbeat and returned to their cages upon recovery. Postoperatively, the rabbits were provided with anti-bite collars to prevent disruption of the surgical incision closure. Penicillin was administered intramuscularly for 3 days post-surgery to prevent infection. Wound healing and the daily activities of the rabbits were promptly monitored (Fig. 3).

Two days post-modeling, signs of redness, heat, and fluctuation on the skin at the modeling site confirmed successful modeling. Subsequently, three rabbits were randomly chosen from Groups B, C, D, and E. Pus samples were aseptically collected from the abscess sites using a sterile needle and inoculated onto Columbia blood agar plates for both aerobic and anaerobic bacterial cultures. After colony growth, individual colonies were isolated, purified, and cultured on separate blood agar plates. Gram staining was performed, and the bacteria were identified using MALDI-TOF MS mass spectrometry.

### Overall Observation and Drainage Volume

Daily assessments will include monitoring wound inflammation, healing progression, obstruction, air leaks, subcutaneous hematoma, mortality, and any associated complications. After 14 days, the rabbits were euthanized, the incision was opened, and the development of granulation tissue on the inner wall was assessed. The weight and skin temperature at the wound site were recorded every morning at 8 a.m. The drainage fluid collected from the negative-pressure reservoir over 24 h was discharged daily at noon, and its color, odor, turbidity, volume, and other characteristics were noted. In addition, the turbidity of the irrigation fluid was evaluated at noon each day.

### In Vitro Comparison of Biocompatibility

#### Live/Dead Cell Staining Experiment

**Fig. 4:**
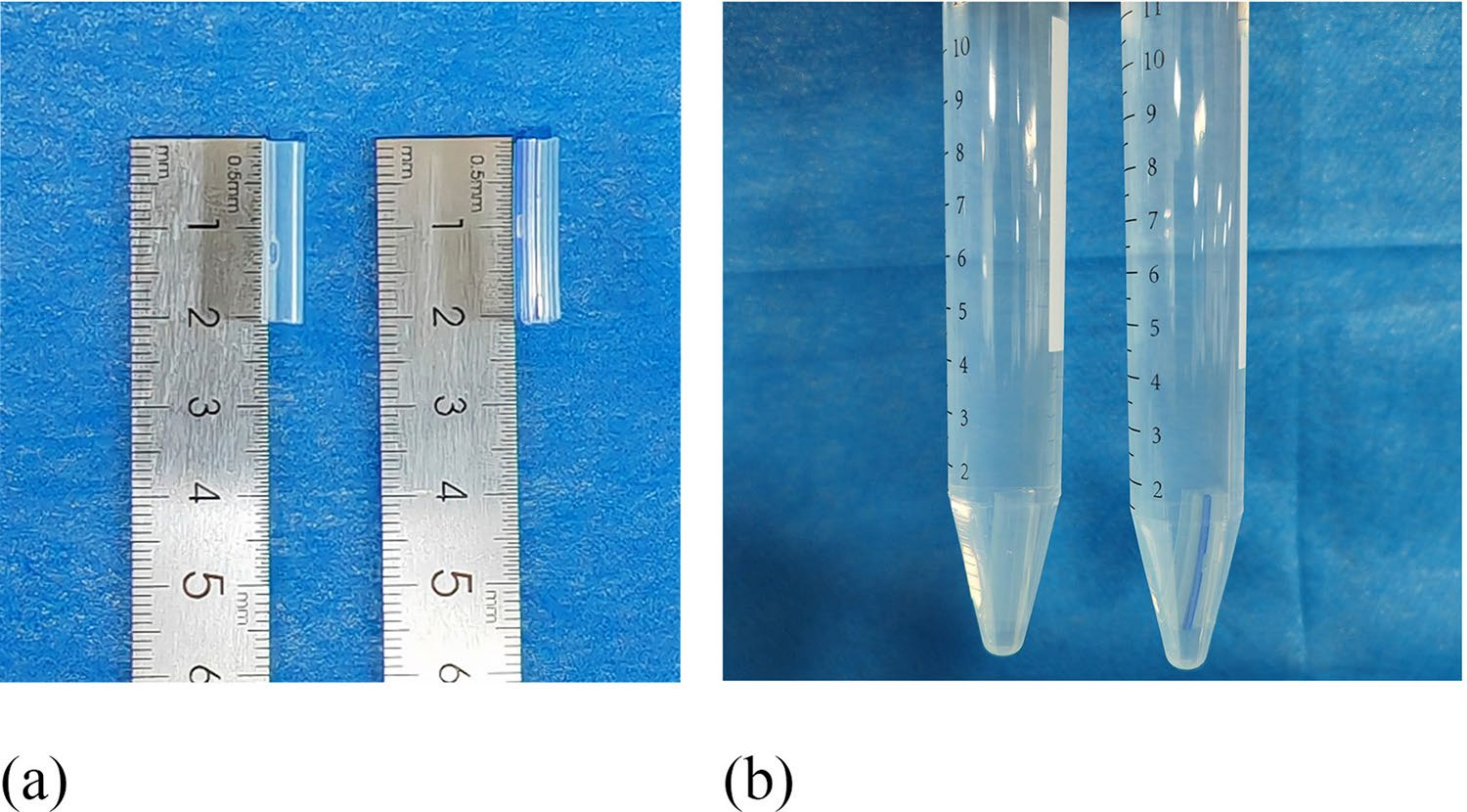
The preparation process of the pan silicone drainage tube and extract of the improved negative-pressure drainage tube; **a** cut pan drainage tube and improved negative-pressure drainage tube 2 cm each; **b** place them under sterile conditions in 5% serum concentration DMEM high-glucose culture medium for 24 h to prepare the extract

The study established three groups: control, pan drainage tube, and improved negative-pressure drainage tube groups. Each pan drainage tube and improved negative-pressure drainage tube was cut to 2 cm in length, which is consistent with the length implanted in the bodies of New Zealand rabbits during animal experiments. After 24 h of sterilization by ultraviolet irradiation, the tubes were immersed in DMEM high-glucose culture medium with 5% serum concentration for 24 h to prepare the extracts (Fig. 4). NIH/3T3 fibroblasts were seeded in six-well cell culture plates at a density of 5 × 10^4^ cells/well. The cells were cultivated in DMEM high-glucose culture medium containing 5% serum, along with the extracts from the pan drainage tube and improved negative-pressure drainage tube, for 24 h, 48 h, and 72 h, respectively. Subsequently, the culture medium was removed and a calcein-AM/PI staining working solution was added following the manufacturer’s instructions. The samples were incubated in a dark cell culture incubator for 15 min, after which the working solution was discarded, and the samples were washed twice with PBS. Cell staining and quantification were performed using an inverted fluorescence microscope with living cells emitting green fluorescence and dead cells emitting red fluorescence.

#### Cell Counting Kit-8

NIH/3T3 fibroblasts were seeded at a density of 5 × 10^3^ cells/well in a 96-well cell culture plate and incubated in a 37 °C constant temperature cell culture incubator; this was repeated in five wells for each group. After 24 h, the culture medium was replaced with 5% serum concentration DMEM high-glucose culture medium, pan drainage tube extract, or improved negative-pressure drainage tube extract, each added separately. Subsequent to the 24-h, 48-h, and 72-h incubation period in the 37 °C, 5% CO2 cell culture incubator, the CCK-8 reagent was introduced for an additional 30 min of incubation, and the absorbance at 450 nm was measured using an enzyme reader.

### Histological Examination

On the 14th postoperative day, New Zealand White rabbits were euthanized, and muscle tissue from the surgical site was harvested and preserved in 4% paraformaldehyde for 15 days. Subsequently, the tissue samples were conventionally embedded in paraffin and sectioned to a thickness of 5 μm. Histological evaluation was conducted using hematoxylin–eosin (HE) and Masson’s staining of these tissue slices. The slides were examined under an optical microscope to assess wound healing.

### Statistical Analyses

All statistical analyses were performed using SPSS Statistics 24.0 (IBM, Armonk, NY, USA). Continuous variables were expressed as mean ± standard deviation or median (interquartile range). Group comparisons were evaluated using one-way analysis of variance for continuous variables, complemented by post-hoc LSD tests for pairwise comparisons. Independent sample t-tests were used for intergroup comparisons. *P* < 0.05 was considered statistically significant.

## Results

### The Measurement of Negative-Pressure Suction Balls

**Fig. 5:**
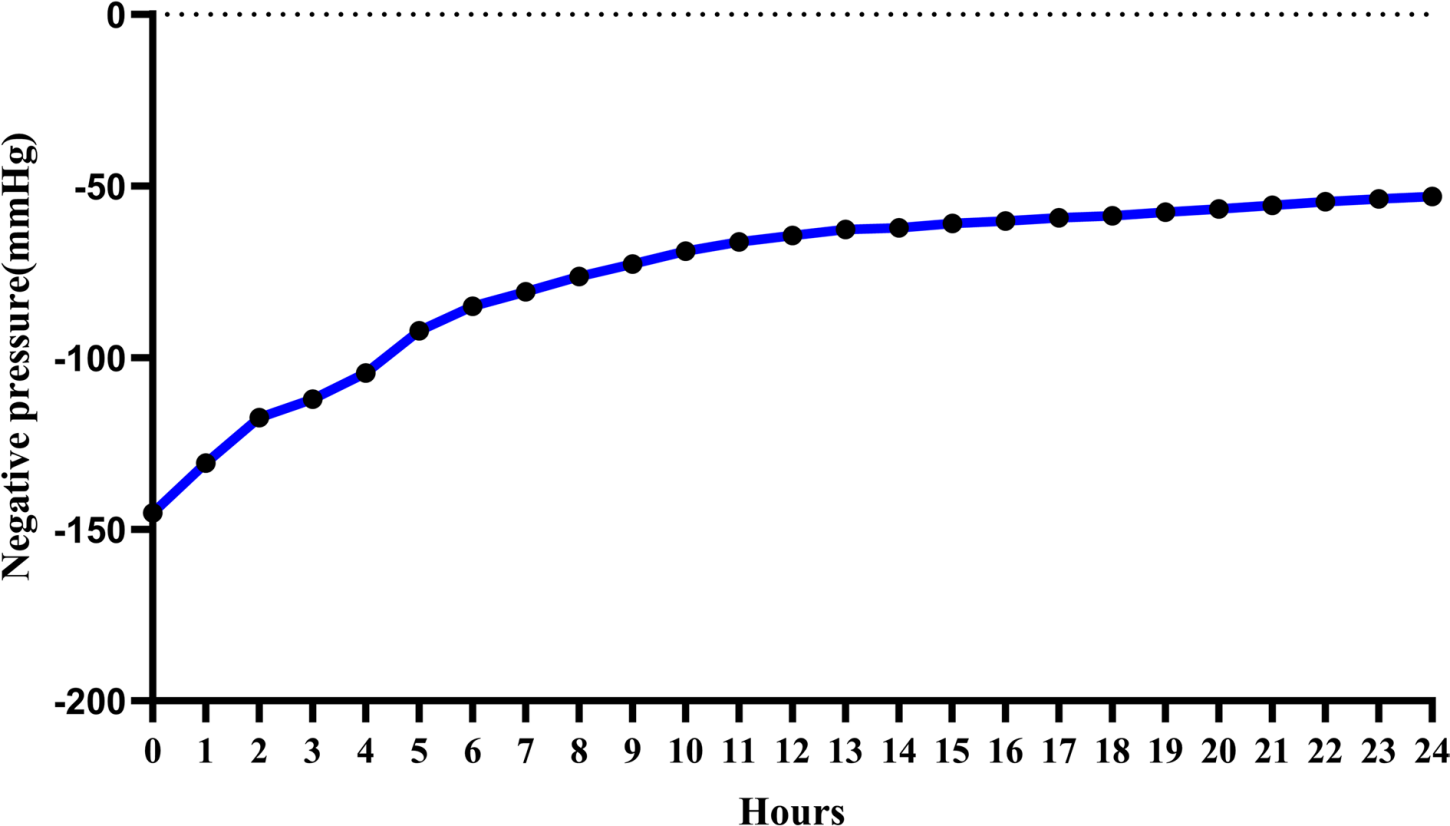
Line graph of pressure changes in the negative-pressure suction balls from full compression to 24 h

Measurements from an electronic manometer indicated that the average negative pressure of three randomly selected negative-pressure suction balls at full deformation is − 145.26 mmHg, and changed to − 53.13 mmHg after 24 h. The median is − 64.41 mmHg, with an interquartile range of (− 88.68, − 58.22) mmHg. The overall pressure exhibited a relatively stable and gradual decline (Fig. 5).

### The Deep Wound Infection Model in New Zealand White Rabbits Was Successfully Established

**Fig. 6:**
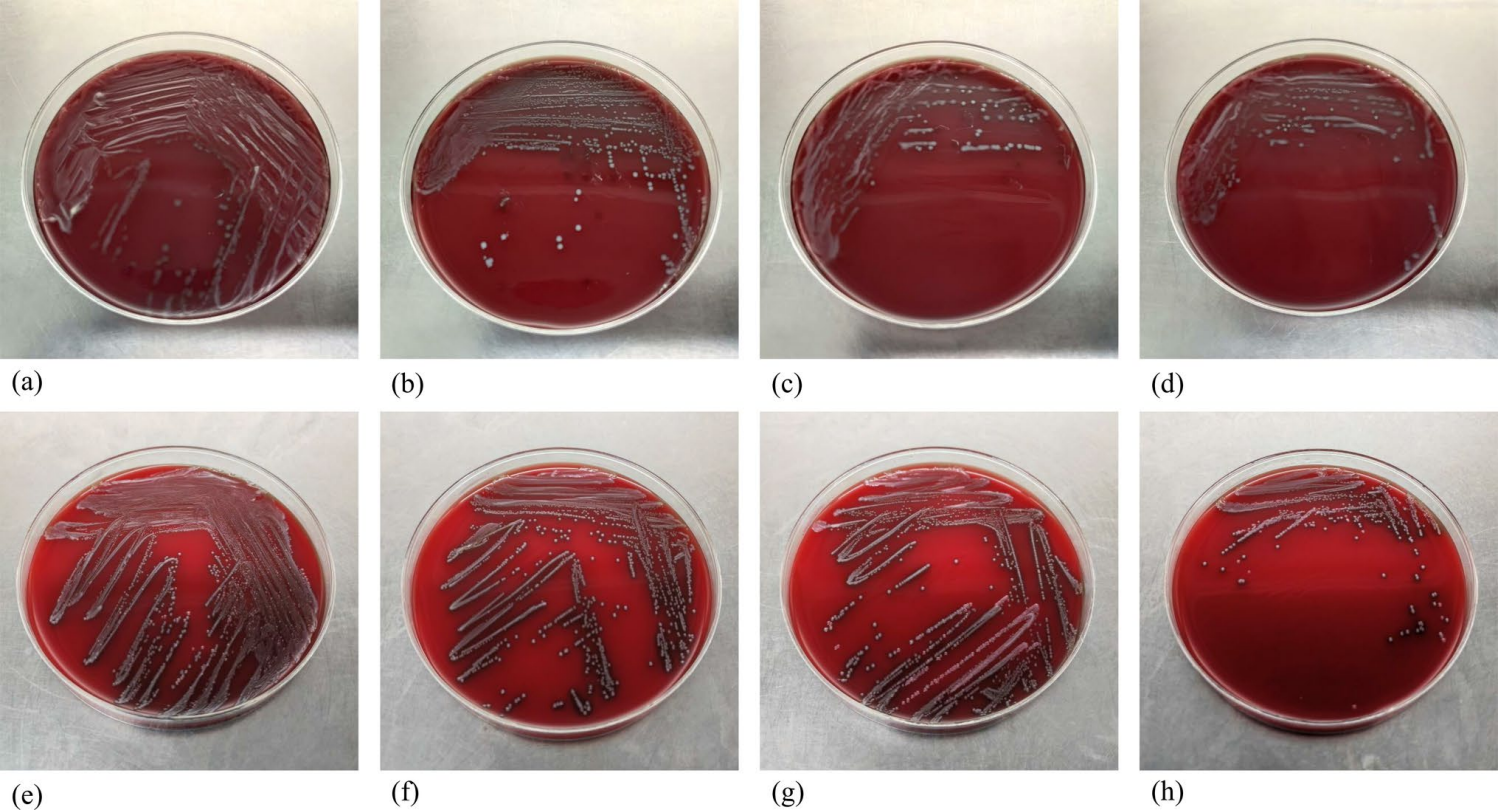
**a** Blood agar plate diagram after anaerobic culture of Group B; **b** blood agar plate diagram after anaerobic culture of Group C;** c** blood agar plate diagram after anaerobic culture of Group D; **d** blood agar plate diagram after anaerobic culture of Group E; **e** blood agar plate diagram after aerobic culture of Group B; **f** blood agar plate diagram after aerobic culture of Group C; **g** blood agar plate diagram after aerobic culture of Group D; **h** blood agar plate diagram after aerobic culture of Group E

**Fig. 7:**
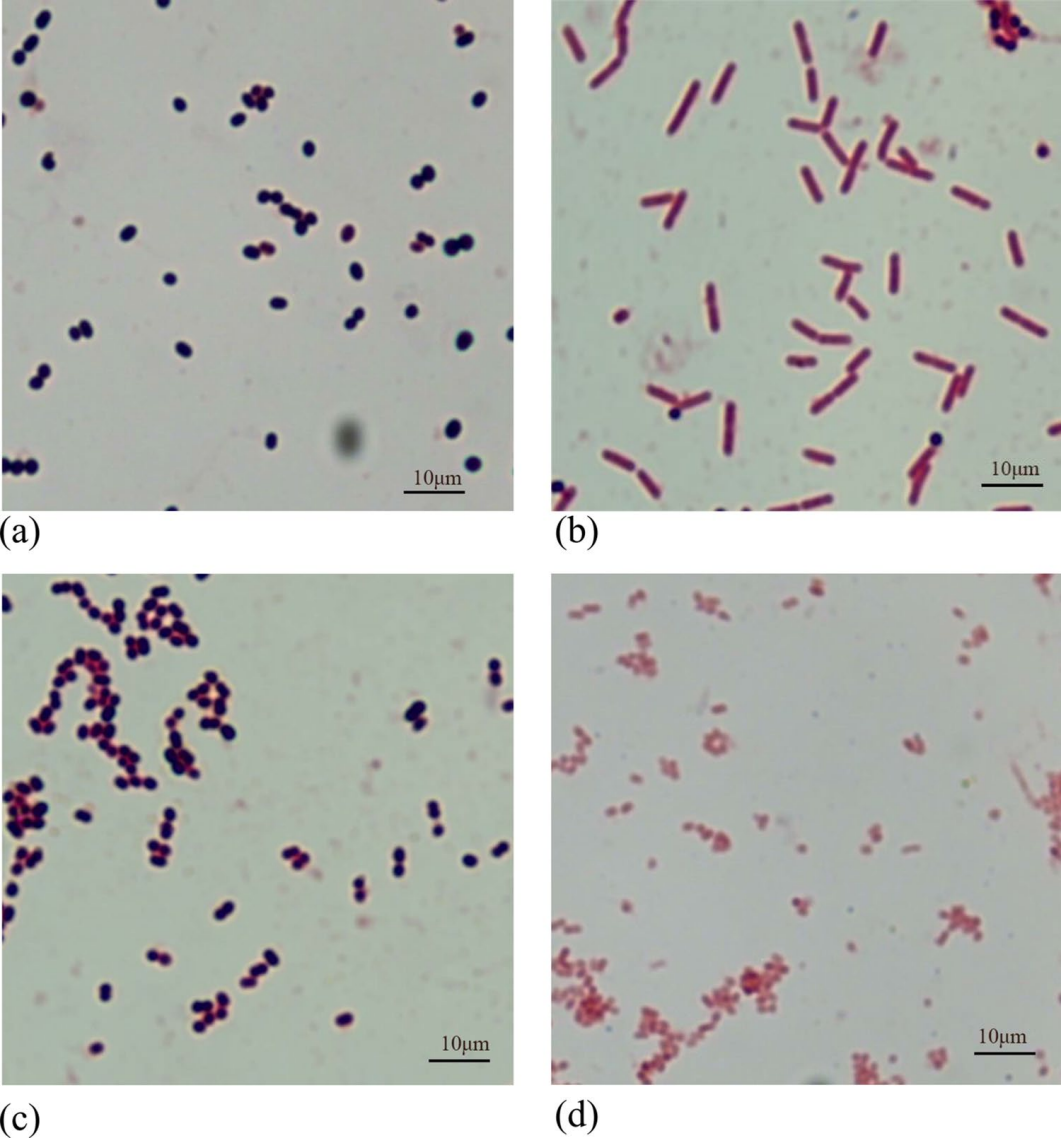
**a** Under the microscope diagram of *Enterococcus faecalis* after anaerobic culture; **b** under the microscope diagram of *Escherichia coli* after anaerobic culture; **c** under the microscope diagram of *Staphylococcus aureus* after aerobic culture; **d** under the microscope diagram of *Acinetobacter baumannii* after aerobic culture

Two days after surgery, a small quantity of pus was aspirated from the abscess site using a sterile needle and inoculated onto Columbia blood agar plates to separate aerobic and anaerobic bacterial cultures. Aerobic colonies developed after 12 h, followed by the growth of anaerobic colonies after 24 h. Distinct colonies were isolated and cultured on individual blood agar plates, followed by Gram staining. The findings revealed that anaerobically cultured bacteria in all four groups consisted primarily of Gram-positive cocci and Gram-negative rods, whereas aerobically cultured bacteria were predominantly Gram-positive and Gram-negative cocci. MALDI-TOF MS identification demonstrated that *Enterococcus faecalis* and *Escherichia coli* were prevalent in anaerobic cultures, whereas *Staphylococcus aureus* and *Acinetobacter baumannii* were dominant in aerobic cultures. The consistency in the major bacterial species identified across Groups B, C, D, and E in pus confirmed the successful establishment of the model (Figs. 6, 7).

### Overall Observation and Drainage Volume

Following successful modeling, the rabbits in Group A maintained normal food intake and activity levels; however, rabbits in Groups B, C, D, and E showed marked reductions. From the 3rd day onward, rabbits in Group B began to exhibit a gradual discharge of purulent material from the wound, whereas rabbits in Groups C, D, and E did not exhibit any purulent discharge at the wound site. By the 8th day, scab formation was observed at the wound site in Groups C and D, and by the 14th day, these wounds had healed completely. In Group E, scab formation commenced on the 6th day, with almost complete healing by the 12th day. Upon euthanizing the rabbits after 14 days, examination of the incisions revealed normal tissue in Group A, whereas Group B exhibited significant retention of purulent fluid at the wound site, accompanied by the presence of a substantial white pus wall and a foul odor. Groups C and D showed evident growth of granulation tissue at the wound site, with minimal white pus wall formation and no odor. In Group E, there was substantial growth of granulation tissue without the formation of a white pus wall. Importantly, none of the rabbit groups experienced complications such as blockage, air leaks, subcutaneous hematoma, or postsurgical mortality at any point in time.

**Fig. 8:**
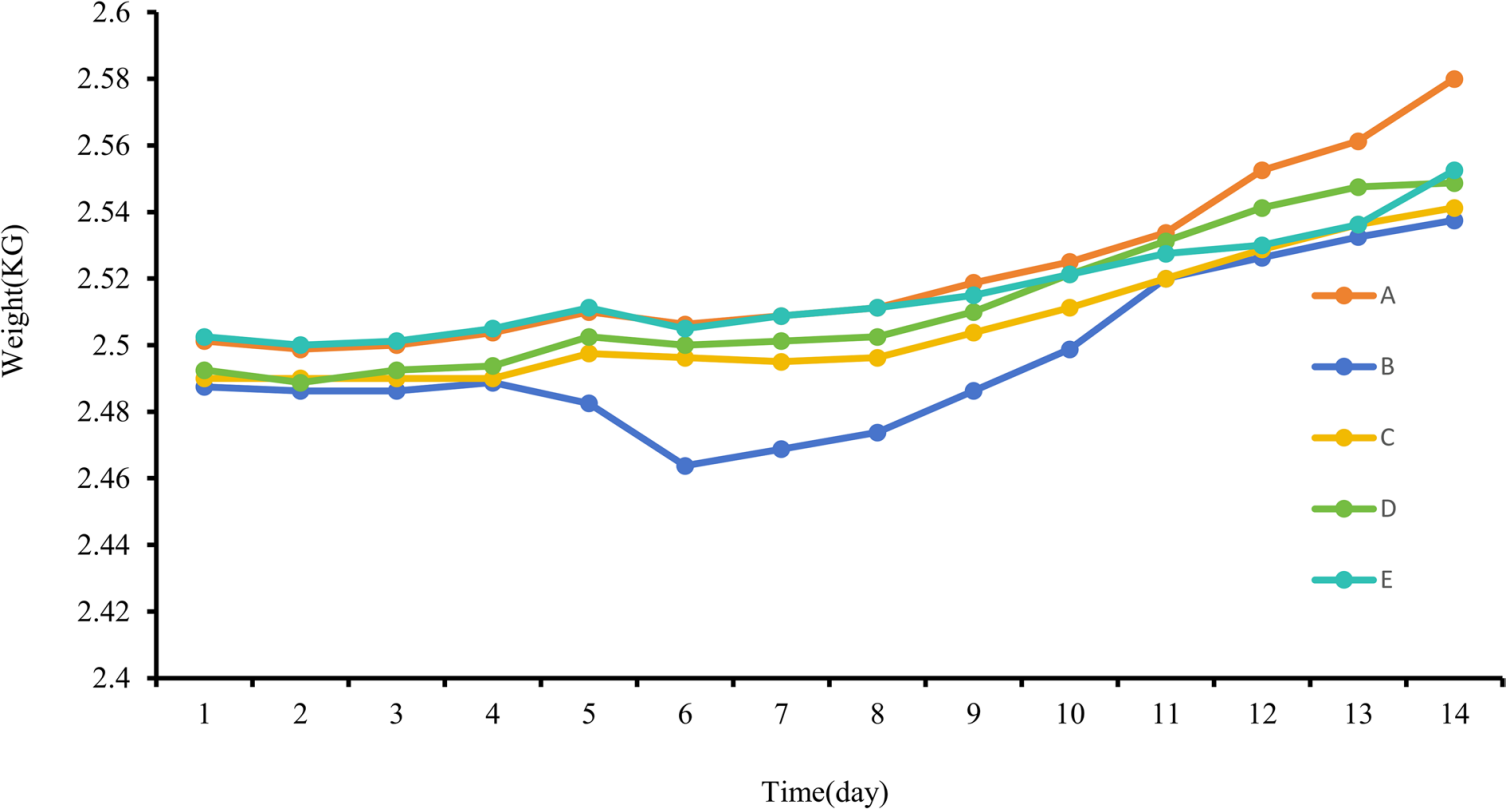
Line chart of daily average weight changes for each group

Following the surgical procedures, the rabbits in Groups A, C, D, and E displayed a gradual increase in body weight. Notably, Group A exhibited the most rapid and substantial weight gain, whereas Groups C, D, and E showed a comparable steady increase, without significant differences. Conversely, Group B rabbits experienced reduced food intake due to the infection, leading to the slowest rate of weight gain and even temporary weight loss (Fig. 8). Statistical analysis revealed no significant variation in weight among the five groups (*P* > 0.05) (Table 2).

**Table 2:** Comparison of average weight among groups after 14 days

Number	*n*	Weight ($\overline{x} \pm s$)/kg	*F*	*P*
A	8	2.52 ± 0.44		
B	8	2.50 ± 0.57		
C	8	2.51 ± 0.51	0.320	0.863
D	8	2.51 ± 0.48		
E	8	2.52 ± 0.54		

**Fig. 9:**
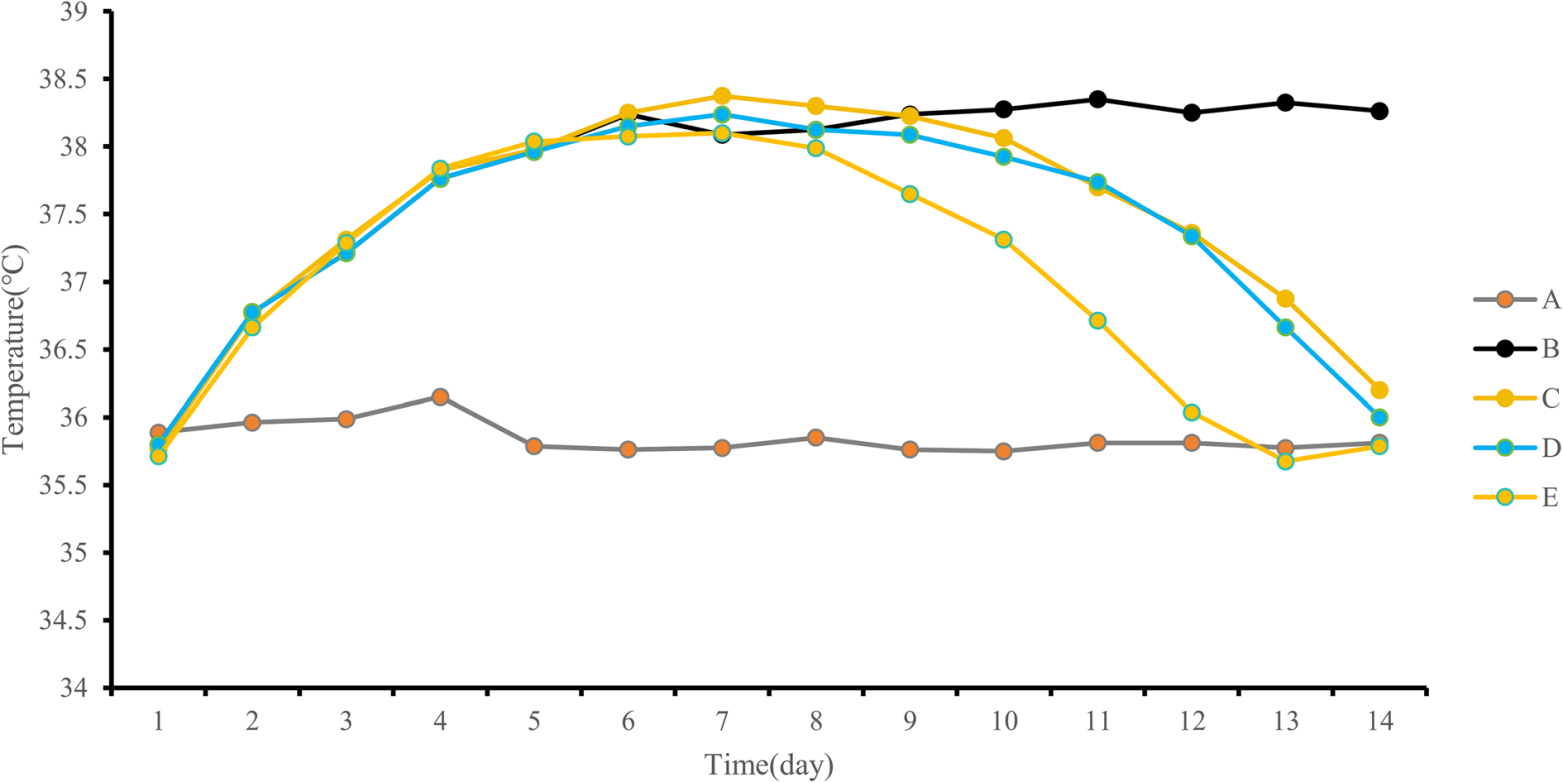
Line chart of daily average skin temperature changes for each group

Skin temperature remained stable in Group A, without significant fluctuations. Groups B, C, D, and E showed an increasing trend in skin temperature from the first postoperative day, reaching a peak between the first and fourth days. Group B exhibited a consistent temperature elevation above 38 °C starting from the 6th day. Conversely, skin temperatures in Groups C and D started declining on the 8th day and eventually returned to preoperative levels by the 14th day. In Group E, the skin temperature started to decrease from the 8th day, reaching preoperative levels by the 12th day (Fig. 9). Statistical analysis revealed substantial temperature variations among the groups (*P* < 0.05). Pairwise comparisons using LSD testing indicated statistically significant differences in skin temperature between any two of the five groups (*P* < 0.05) (Table 3).

**Table 3:** Comparison of average skin temperature among groups over 14 days

Number	*n*	Skin temperature ($\overline{x} \pm s$)/℃	*F*	*P*
A	8	35.85 ± 0.72		
B	8	37.83 ± 0.78		
C	8	37.50 ± .0.59	1171.525	< 0.001
D	8	37.41 ± 0.59		
E	8	37.06 ± 0.45		

**Fig. 10:**
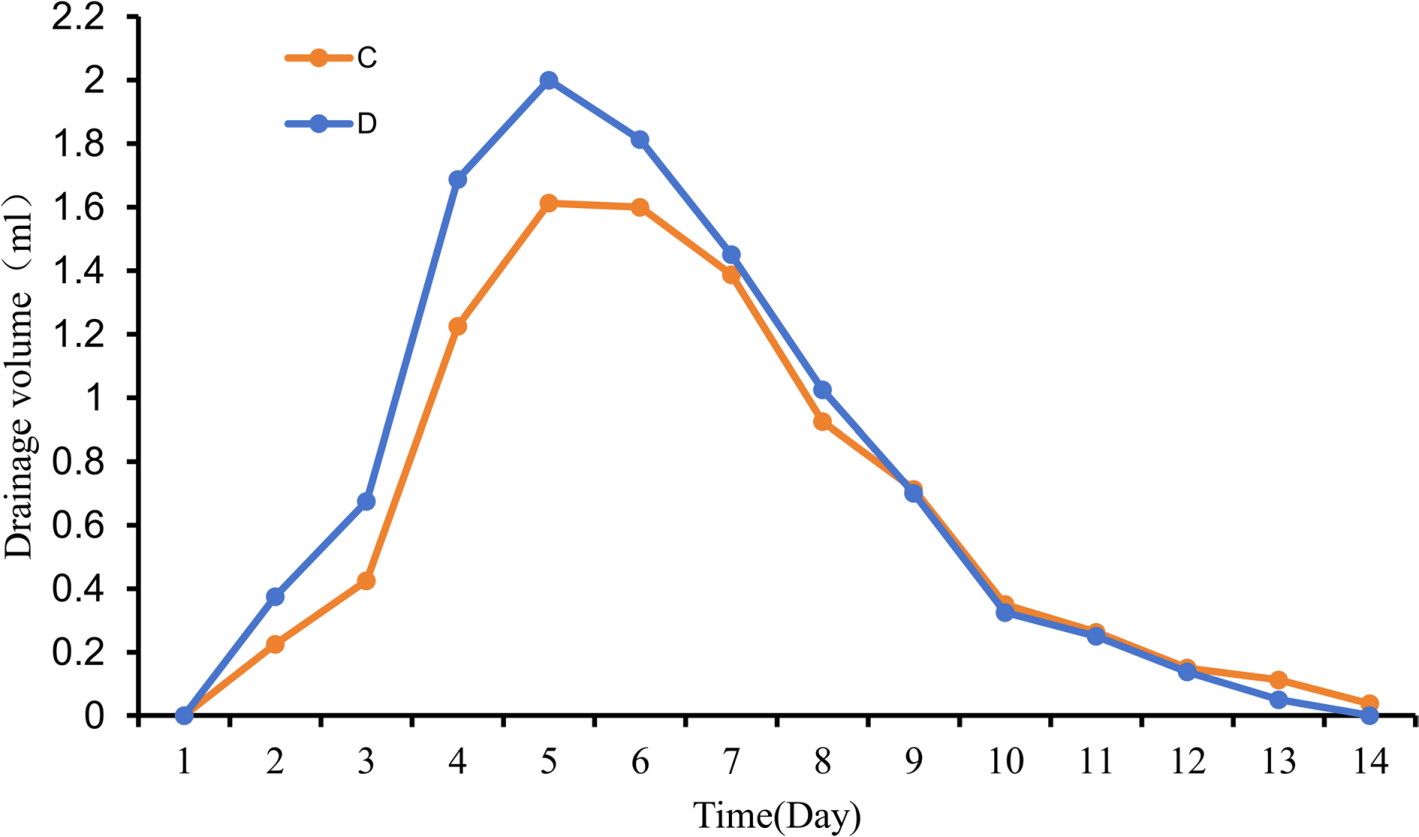
Line graph showing the daily average drainage volume changes in each group

On the second postoperative day, Groups C and D underwent pus aspiration, which yielded a milky-yellow turbid liquid with a putrid odor and flocculent sediment upon settling. The rabbits showed varying daily drainage volumes, ranging from 0.1 ml to 2.2 ml. Both groups displayed an initial increase, followed by a decrease in the daily average drainage volume, which peaked on the 5th day. Between the 1st and 8th days, Group D had a higher daily average drainage volume than Group C. However, from the 9th day onward, Group C exhibited a higher daily average drainage volume. This suggests that Group D initially experienced more comprehensive drainage, resulting in a subsequent decline in the daily drainage volume (Fig. 10). In this study, over 14 days, the total average drainage volume was 9.03 ± 0.60 ml for Group C and 10.49 ± 0.99 ml for Group D, illustrating a higher drainage volume in Group D. Statistical analysis revealed a significant difference in drainage volume between the two groups (*P* < 0.05) (Table 4).

**Table 4:** Comparison of the average total drainage volume over 14 days in each group

Number	*n*	Drainage volume ($\overline{x} \pm s$)/ml	*t*	*P*
A	8	/		
B	8	/		
C	8	9.03 ± 0.60	− 3.569	0.003
D	8	10.49 ± 0.99		
E	8	/		

**Fig. 11:**
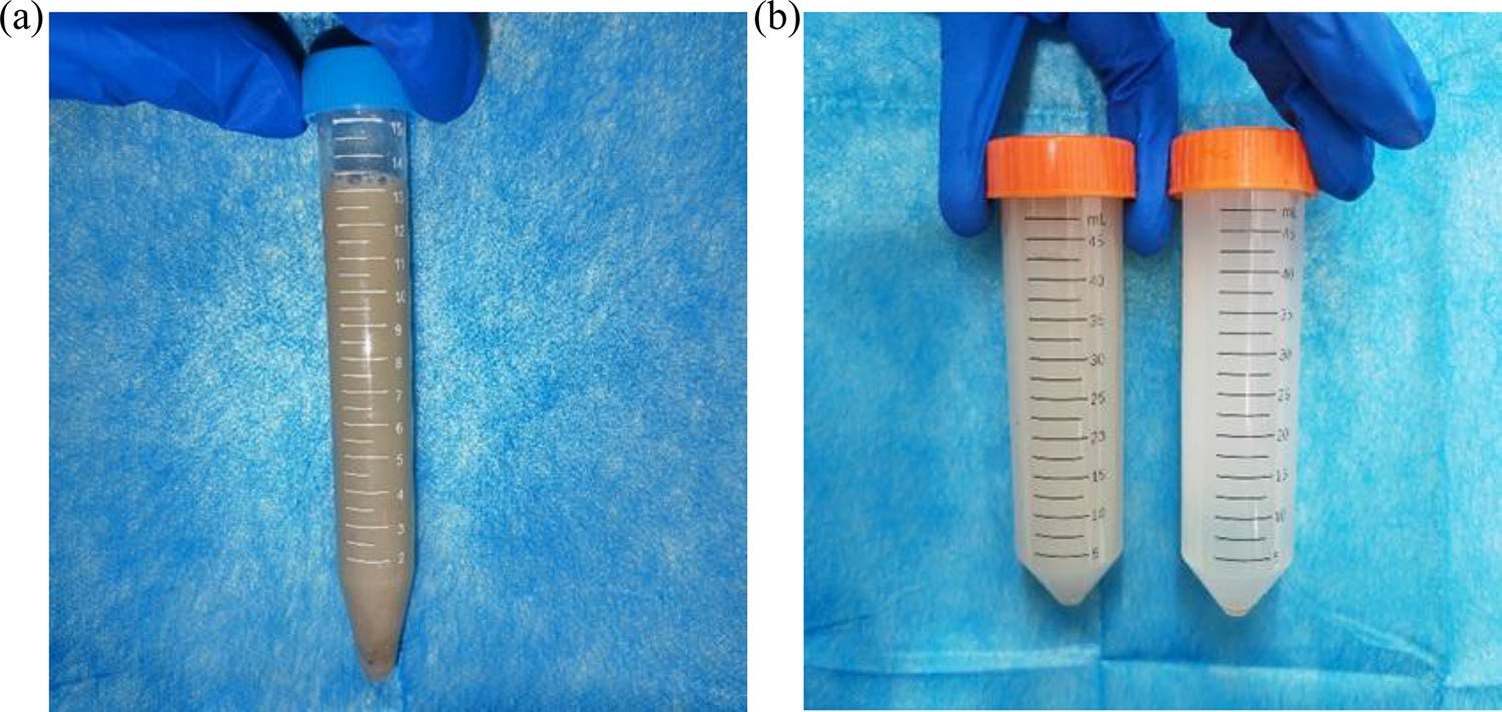
**a** Diagram of pus; **b** left side shows a diagram of the flushing fluid on the 5th day after surgery, right side shows a diagram of the flushing fluid on the 10th day after surgery

In addition, the liquid flushed out in Group E became clearer over time, indicating that with daily flushing, there was less pus accumulation at the infected site, and the wound healing status gradually improved (Fig. 11).

### In Vitro Comparison of Biocompatibility

**Fig. 12:**
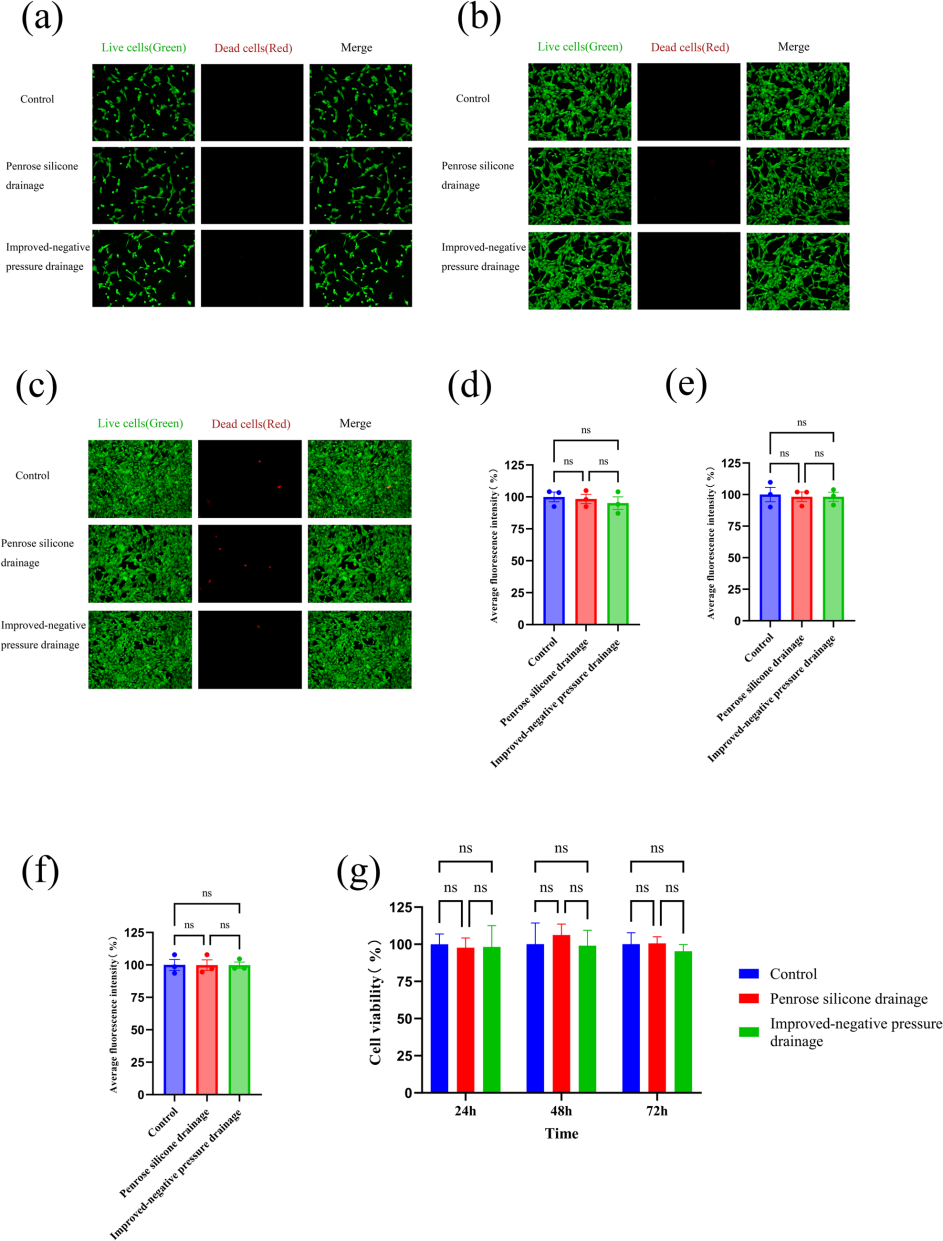
Schematic diagrams of live and dead cell staining of NIH/3T3 fibroblast cells at 24/48/72 h (**a**–**c**), average immunofluorescence intensity analysis of live and dead cell staining of NIH/3T3 fibroblast cells at 24/48/72 h (**d**–**f**), and analysis of CCKit-8 results of NIH/3T3 fibroblast cells (**g**) in the control, pan silicone drainage tube group, and improved negative-pressure drainage tube groups at 24/48/72 h

The biocompatibility of the enhanced negative-pressure drainage tube was assessed through in vitro experiments. Cell staining revealed that the control, pan drainage tube, and improved negative-pressure drainage tube groups exhibited abundant viable (green) and minimal dead (red) cells after 24 h, 48 h, and 72 h of culture. Subsequent analysis of the average fluorescence intensity showed no substantial detrimental effect on cell proliferation across the three groups, and no significant statistical variance was observed. Subsequently, we employed a CCK-8 assay kit to evaluate the cytotoxicity of the pan silicone drainage tube and extract from the improved negative-pressure drainage tube on NIH/3T3 fibroblast cells. After incubation, the viability of NIH/3T3 fibroblast cells in the control, pan drainage tube, and improved negative-pressure drainage tube groups remained largely unaffected after 24 h, 48 h, and 72 h of culture, with no significant statistical variations (Fig. 12).

### HE Staining

**Fig. 13:**
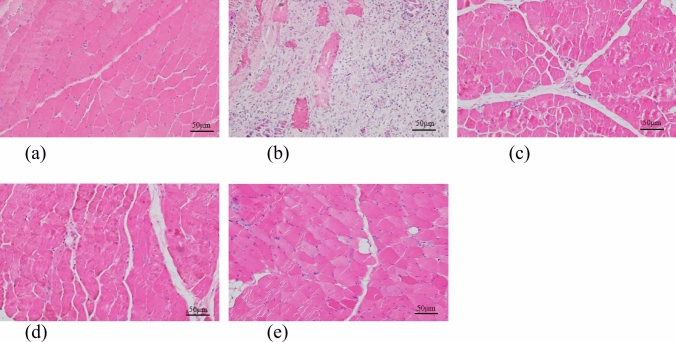
HE staining results of muscle specimens in each group (× 200)

On the 14th postoperative day, New Zealand White rabbits were euthanized, and muscle tissue from the deep wound site was subjected to Hematoxylin and Eosin (HE) staining. The findings revealed that in Group A, muscle tissue exhibited a normal structure characterized by smooth muscle fiber surfaces, consistent spacing between muscle bundles, well-organized muscle cells of uniform size, clear cell boundaries, absence of degeneration, and no noticeable fibrosis or infiltration of inflammatory cells. In contrast, Group B displayed markedly aberrant muscle tissue with a disrupted cell structure, pronounced fibrosis, extensive formation of fibrous tissue, heightened proliferation of inflammatory cells, evident morphological deformities, enlargement of neighboring muscle cells, and widespread vacuolization of muscle cells, indicative of severe infection. Group C manifested a slightly abnormal muscle tissue structure characterized by indistinct muscle fibers, minimal lymphocyte infiltration, mild enlargement and vacuolization of muscle cells, and limited necrotic cell fragments. Similarly, Group D exhibited a mild abnormality in the muscle tissue structure, with a relatively uniform arrangement of muscle cells, mild fibrosis in specific regions, slight development of fibrous tissue, and minimal proliferation of inflammatory cells. Conversely, Group E displayed a predominantly normal muscle tissue structure, characterized by uniform cell size and consistent spacing between muscle bundles, and exhibited no significant structural deviations compared to Group A (Fig. 13).

### Masson’s Staining

**Fig. 14:**
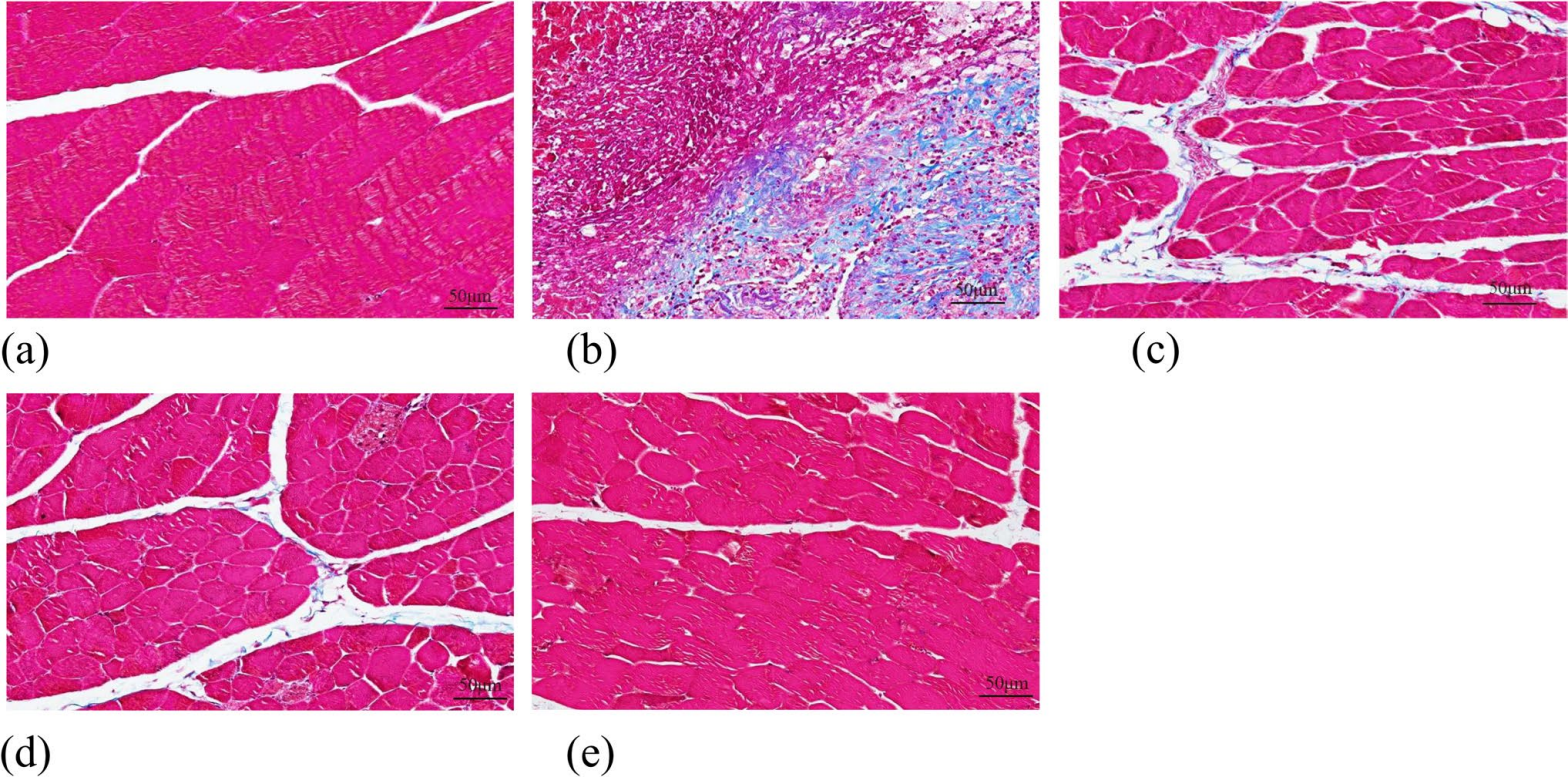
Masson staining results of muscle specimens in each group (× 200)

On the 14th postoperative day, New Zealand White rabbits were euthanized, and muscle tissue from the deep wound site was subjected to Masson’s staining. Masson’s trichrome staining revealed that mature reticular collagen fibers, cytoplasmic matrix, and muscle fibers were stained red, whereas new collagen fibers and cartilaginous tissue appeared blue. The findings revealed that Group A did not show any noticeable deposition of blue collagen fibers in the muscle interstitium. In Group B, the majority of collagen fibers were degraded, likely due to the action of collagenase produced by the bacteria. Both Groups C and D exhibited limited blue collagen fibers in the muscle interstitium, with Group C displaying a larger area of blue staining, indicating increased collagen fiber deposition. Group E demonstrated a relatively well-organized structure with no significant deviations from Group A (Fig. 14).

## Discussion

The primary outcomes of this study indicate that the enhanced negative-pressure wound irrigation drainage tube can efficiently facilitate seamless drainage of deep wounds in animal models, markedly ameliorate the extent of wound infections and inflammatory infiltration, and ultimately enhance overall wound healing. Utilizing an improved negative-pressure wound irrigation drainage tube, in conjunction with normal saline flushing, can lead to enhanced therapeutic efficacy.

Previous research has indicated that deep infection is a prevalent and severe complication of orthopedic surgeries. A retrospective study covering 9 years involving 90,551 patients who underwent spinal surgery reported an infection rate of 1.4% at the surgical site [15]. This is primarily attributed to the common use of implants in orthopedic procedures, which significantly increases the risk of infection [2, 3]. Deep wound infections often present with atypical clinical signs and can result in substantial severity, potentially leading to systemic infection, and multi-organ failure, and posing a threat to patient safety. Hence, enhancing the accurate diagnosis and treatment of deep wound infections after orthopedic surgery is paramount for enhancing patient outcomes [16]. Presently, the primary modalities for managing deep wound infections after orthopedic surgery include debridement and antibiotic therapy, albeit with inherent constraints. Overprescription in medical settings and self-medication practices may foster drug misuse, increasing the likelihood of recurrent infections, antibiotic resistance, and other complications, thereby compromising clinical treatment efficacy [5, 6, 17].

Research findings indicate that excessively high and low negative pressures can have adverse effects. Extremely high negative pressure, such as − 400 mmHg, may excessively compress blood vessels, resulting in damaged subcutaneous tissues [18]. Conversely, extremely low negative pressures, ranging from − 25 to − 30 mmHg, can impede fluid drainage, thereby affecting the wound healing process. Generally, the range of − 50 to − 175 mmHg is deemed more appropriate [19]. Here, we initially randomly selected three negative-pressure suction balls from one batch, connected them to an electronic manometer, and monitored the negative-pressure fluctuations from full compression to 24 h. The findings revealed that the average negative pressure of the suction balls at full deformation and 24 h was − 145.26 mmHg and − 53.13 mmHg, respectively, both falling within a reasonable range of negative pressure. This indicates the alignment of our study with clinical scenarios, and the results exhibited a certain degree of precision and impartiality. Furthermore, a uniform method was employed to induce modeling in all experimental animals. Two days after the operation, a small amount of pus was aspirated from the abscess site using an empty needle and cultured on Columbia blood agar plates for aerobic and anaerobic bacterial cultures. Subsequently, Gram staining and MALDI-TOFMS mass spectrometry were performed. The results revealed consistently predominant bacterial species across all the groups, including *Staphylococcus aureus*, *Enterococcus faecalis*, *Escherichia coli*, and *Acinetobacter baumannii*. Garcia et al. reported that *Staphylococcus aureus* was the most prevalent pathogen in spine, elbow, hand, and ankle infections, with *Enterococcus faecalis* and *Escherichia coli* commonly implicated in postoperative orthopedic infections [20]. This suggests that our animal model closely mirrors actual clinical situations, resulting in informative outcomes.

In recent years, NPWT has been widely used in clinical practice owing to its ability to promptly remove wound exudates, promote granulation tissue growth, and facilitate wound healing. However, it still faces certain challenges [21]. Currently, the commonly used negative-pressure drainage tubes are mostly single-lumen and relatively soft, often leading to inadequate drainage and retention. Moreover, single-lumen tubes have limited functionality because they cannot simultaneously perform irrigation, medication delivery, and other procedures, resulting in inconvenience. Furthermore, conventional negative-pressure drainage tubes are difficult to place in deep wounds and are challenging to visualize in many imaging studies, making it difficult for clinicians to accurately assess their positioning [22, 23].

The findings from this study revealed that the average body temperature of rabbits in groups D and E, which utilized the enhanced negative-pressure wound irrigation drainage tube, was significantly lower than that of the other three groups at 14 days (*P* < 0.001). This is attributed to the construction of the tube from silicone rubber, which is known for its high compatibility with human tissues, thereby reducing the likelihood of rejection reactions and minimizing the impact of drainage tube insertion on rabbits. Moreover, during the drainage process, group C had an average total drainage volume of 9.03 ± 0.60 ml at 14 days, while group D exhibited 10.49 ± 0.99 ml, demonstrating that group D had a greater volume than group C (*P* < 0.05). These results suggest that the improved drainage tube, with its unique four-edge-supporting plum blossom-shaped cross-section, effectively prevents blockages during drainage, thereby ensuring a smoother process. Research by Li et al. in a pig infective tissue damage model confirmed that NPWT stimulates granulation tissue growth and accelerates wound healing better than traditional gauze dressings [13]. In this study, group C displayed minor abnormalities in the muscle tissue structure, characterized by indistinct muscle fibers, reduced necrotic cell fragments, and inflammatory infiltrates. In contrast, group D exhibited a relatively organized arrangement of muscle cells, with marginal fibrosis in certain areas and subtle fibrous tissue formation. Correspondingly, under Masson staining, both groups C and D presented a minimal presence of blue collagen fibers in the muscle interstitium, whereas group C displayed a larger area of blue staining, indicating a higher deposition of collagen fibers. The outcomes of both staining methods suggest that the enhanced negative-pressure wound irrigation drainage tube can more efficiently alleviate wound inflammation infiltration, providing substantial benefits for wound care and promoting healing. Compared to KCI’s efficient NPWTi, our enhanced negative-pressure drainage tube is more cost-effective, benefiting patients with limited financial resources. In addition, the simplified operation of the improved drainage tube negates the need for intricate parameter adjustments, ensuring ease of use in the placement and drainage procedures, which are particularly suitable for healthcare facilities in resource-limited settings. Moreover, the lightweight design allows patients to be discharged with the device for home use under medical supervision, enhancing convenience and accessibility for patients.

Although NPWT is extensively used in clinical settings, the comparative efficacy of negative-pressure wound therapy with instillation (NPWTi) and traditional NPWT remains debatable. A study by Omar revealed that NPWTi could notably shorten hospital stays and expedite wound healing compared with standalone NPWT, despite statistical findings showing no significant disparity between the treatment approaches [24]. Conversely, in the treatment of a patient with a lower limb soft tissue injury, Ali observed that standalone NPWT did not significantly accelerate wound healing, while NPWTi stimulated rapid granulation tissue growth at the wound site, even covering the exposed bone [25]. This observation is further supported by studies conducted by Timmers and Jukema [26, 27]. In the current study, group E, which utilized an enhanced negative-pressure wound irrigation drainage tube with saline irrigation, exhibited the earliest formation of wound scabs on postoperative day six. Upon euthanization 14 days postoperatively, significant granulation tissue growth was evident at the wound site in Group E, with no formation of a white purulent wall. In addition, the average body temperature of rabbits in group E was the lowest among the five groups at 14 days (37.06 ± 0.45 °C, *P* < 0.001), indicating the group’s optimal stability. Biocompatibility is a crucial criterion for evaluating implanted materials in the body. Cell staining of live and dead cells and CCKit-8 assays confirmed that the control, pan silicone drainage tube, and enhanced negative-pressure drainage tube groups demonstrated abundant viable (green) and minimal dead (red) cells. Furthermore, after 24 h, 48 h, and 72 h of culture, neither the pan silicone drainage tube nor extract from the improved negative-pressure drainage tube significantly affected the activity of the NIH/3T3 fibroblast cells. These results indicate that our product exhibits favorable biocompatibility at the cellular level. Furthermore, HE and Masson staining confirmed that the muscle tissue structure in group E was largely normal, with uniform cell sizes and no significant deviations compared to healthy tissues. These results highlight the potential of NPWTi to deliver continuous irrigation, consecutively remove exudates and necrotic tissue, efficiently manage local infections, and enhance wound healing, thus offering promise for the treatment of more complex and challenging wounds in the future.

The limitations include: (1) small sample size. (2) Skin mobility in the rabbit model of deep wound infection was significantly greater than that in humans, complicating the full simulation of orthopedic wounds. (3) Despite the confirmed benefits of using rabbits as a model for randomized controlled trials, there are differences from humans that may potentially affect postoperative recovery. This animal study validated the clinical advantages and effectiveness of the improved negative-pressure wound irrigation drainage tube, providing supporting evidence for its future clinical applications and introducing new, improved options for the use of NPWT and NPWTi.

In conclusion, the modified negative-pressure wound irrigation drainage tube has definite advantages over the traditional Penrose silicone drainage tube in terms of improving deep wound inflammatory infiltration, optimizing wound treatment, and promoting healing.

## Conclusion

Findings from animal experiments, cell studies, and histological investigations indicated that the enhanced negative-pressure wound irrigation drainage tube, compared to the conventional pan silicone drainage tube, does not exhibit a discernible adverse effect on cellular activity and growth. Moreover, it offers notable benefits for alleviating wound inflammation, enhancing wound management, and fostering healing. Importantly, the combination of an improved negative-pressure wound irrigation drainage tube with physiological saline irrigation is an enhanced approach for facilitating the recovery of infected wounds.

## Data Availability

The datasets used and/or analyzed during the current study available from the corresponding author on reasonable request.
